# Urgent need for services to support children with and at risk of developmental disabilities in low‐ and middle‐income countries

**DOI:** 10.1111/dmcn.16503

**Published:** 2025-09-09

**Authors:** Nathaniel Scherer, Cally J Tann, Hannah Kuper

**Affiliations:** ^1^ International Centre for Evidence in Disability London School of Hygiene & Tropical Medicine London UK; ^2^ The Centre for Maternal, Adolescent, Reproductive, and Child Health London School of Hygiene & Tropical Medicine London UK

## Abstract

**This commentary is on the original article by Katangwe‐Chirwa et al. on pages** 651‐660 **of this issue.**

The Global Burden of Disease Study estimates that there are 53 million children under 5 years of age with developmental disabilities worldwide, with prevalence higher in low‐ and middle‐income countries (LMICs).^1^ However, there is great uncertainty in this estimate because of lack of primary data. Katangwe‐Chirwa et al.'s publication therefore makes an important contribution by estimating cerebral palsy (CP) prevalence and causes in an LMIC.^2^ They demonstrate that CP is common, and often occurs due to preventable neonatal risk factors, such as birth asphyxia, preterm birth, and neonatal infection.

Katangwe‐Chirwa et al.'s paper highlights the need to improve access to timely and high‐quality neonatal care to reduce the occurrence of CP and other developmental disabilities.^3^ Antenatal interventions, such as maternal immunization, nutritional supplementation, infection screening, and risk‐based obstetric care can reduce the incidence of adverse perinatal events. Intrapartum strategies, including fetal monitoring and emergency obstetric capacity, can prevent birth‐related neurological injury. Postnatal examination and care, including optimal care of preterm and low birthweight infants, can prevent morbidity after birth.

However, support is also needed for the children living with developmental disabilities, including children with CP, as they face significant inequalities, including poverty, social exclusion, limited participation, and violence. Evidence shows that access to early intervention is vital to improve functional outcomes and quality of life for children with disabilities.^4^ The first step is to improve early identification of children at developmental risk or with developmental delay, in order to provide timely developmental support to promote positive outcomes. Developmental surveillance, particularly in the first 2 years of life, is essential to allow for early identification. It requires culturally relevant and age‐appropriate developmental assessment tools, which are now increasingly available and validated for low‐resource settings, including the Malawi Development Assessment Tool and the Kilifi Developmental Inventory. Yet, access to early identification services remains limited for many children and families, especially in LMICs, and must be integrated and scaled into routine care.

The second need is for early, targeted support for children with or at high risk of developmental disabilities, as this can enhance developmental trajectories and improve child participation at home and in the community. Early intervention approaches also provide psychosocial and practical support to caregivers, mitigating risks arising from exclusion, stigma, and limited knowledge on disability. In LMICs, however, early intervention services are often scarce, located in urban areas, and under‐resourced. Models of care are needed that are scalable and contextually appropriate.

Family‐centred, community‐based programmes can help to fill the gap in services. Examples include Ubuntu, Baby Ubuntu, and the World Health Organization's (WHO) Caregiver Skills Training Programme, designed for resource‐limited settings. These participatory, group‐based interventions integrate caregiver education, peer support, and practical strategies to promote child development and inclusion. Such early intervention strategies can complement health system functions by linking families to medical, social, and educational resources. They can also promote community awareness on disability and disability rights. High‐quality evidence is needed on the impact of these programmes in LMICs, which are underway in several settings, including the SPARK trial of the WHO Caregiver Skills Training Programme in Kenya and Ethiopia, and the Paediatric Development Clinic/Baby Ubuntu trial in Rwanda.

Katangwe‐Chirwa et al.'s publication makes a critical contribution to the literature by highlighting the need for resources and strategies to improve neonatal care and promote early childhood development for children with and at risk of developmental disability. However, global funding schemes are currently inadequate to address the needs of this population^5^ and the situation is likely to worsen with cuts to global funding. Katangwe‐Chirwa et al.'s paper has demonstrated need – we must now collaborate across disciplines to implement evidence‐based solutions and support all children to thrive.

## Data Availability

Not required.
